# Recent trends in the incidence and survival of stage I liver cancer: a surveillance, epidemiology, and end results analysis

**DOI:** 10.1080/07853890.2022.2131328

**Published:** 2022-11-12

**Authors:** Xue-Chen Yu, Ji-Bin Liu, Qing-Hai Tang, Xun Diao, Qi-Yu Fan, Zhong-Yan Huang, Xiao-Mei Tang, Sha Li, Yong-Feng Cao, Yu-Shui Ma, Da Fu

**Affiliations:** a Institute of Oncology, Affiliated Tumor Hospital of Nantong University, Nantong, China; b Department of Mathematics, Statistics, and Computer Science, Macalester College, Saint Paul, MN, USA; c Hunan Key Laboratory for Conservation and Utilization of Biological Resources in the Nanyue Mountainous Region and College of Life Sciences and Environment, Hengyang Normal University, Hengyang, China; d General Surgery, Ruijin Hospital & Institute of Pancreatic Diseases, Shanghai Jiaotong University School of Medicine, Shanghai, China; e Department of Oncology, Affiliated Tumor Hospital of Nantong University, Nantong, China; f Cancer Institute, Longhua Hospital, Shanghai University of Traditional Chinese Medicine, Shanghai, China; g Central Laboratory for Medical Research, Shanghai Tenth People’s Hospital, Tongji University School of Medicine, Shanghai, China

**Keywords:** HCC, stage, SEER, treatment, prognosis

## Abstract

**Background:**

Improvements in screening and imaging technologies and treatment of liver disease have influenced the trend in diagnosis for stage I liver cancer. In this article, recent trends in age, incidence, tumour size, and survival of different stages of liver cancer are analysed.

**Methods:**

Surveillance, Epidemiology, and end results data from the National Cancer Institute were used to analyse trends in age-adjusted incidence rate, mean tumour size at diagnosis, age at diagnosis, and 5-year survival probability for stage I liver cancer.

**Results:**

Stage I cases of liver cancer increased most tremendously over the study period, with a greater increase from 2004 to 2012 following a smaller increase from 2012 to 2015. Moreover, the mean age of stage I liver cancer increased by 1.72 years from 2004 to 2015. The 5-year-overall survival for stage I liver cases worsened from 97.9% to 83.7% from 2004 to 2011, whereas the 10-year survival probability for stage I cases worsened from 97.3% in 2004 to 79.6% in 2006. Comparing with higher stage cases, stage I liver cancer were more likely to be females, be married, live in metro areas, receive chemotherapy, and carry medical insurance.

**Conclusions:**

The incidence of stage I liver cancer has increased over the study period, with an increase in age of diagnosis, decrease in tumour size, and generally stable overall survival rate with slight decrease. These trends emphasized the importance of early detection of liver cancer and regular screening and better treatment for high-risk populations.

**RESEARCH HIGHLIGHTS**
- Improvements in screening and imaging technologies and treatment of liver disease have influenced the trend in diagnosis for liver cancer.
- Stage I cases of liver cancer increased most tremendously over the study period, with a greater increase from 2004 to 2012 following a smaller increase from 2012 to 2015.
- These trends emphasized the importance of early detection of liver cancer and regular screening and better treatment for high-risk populations.

## Introduction

Liver cancer is one of the most frequent fatal malignancies worldwide [1] causing 830 000 deaths in 2020 [2]. The incidence of liver cancer demonstrates an increasing trend in developed countries and remains the eighth most common malignancy in women and fifth in men [3].

The death rates of liver cancer have been increasing faster than any other cancer [4]. The prognosis of liver cancer is very poor, unless it is caught early [5]. Diagnosis of liver cancer at early stages has proved to lead to better prognosis. Thus, diagnosing stage I liver cancer is crucial. Since stage I liver cancer can often be asymptomatic, regular screening, and surveillance of the high-risk population is helpful and can improve survival probability [6, 7]. Liver cancer is likely caused by underlying diseases, including hepatitis B virus, hepatitis C virus, and fatty liver disease [8]. Other factors such as alcohol consumption, type 2 Mellitus, and smoking are also risk factors [9].

We hypothesize that early detection of liver cancer can lead to a better prognosis and increase in survival probability. Data from the surveillance, epidemiology and end results (SEER) was used to analyse the incidence rates, age at diagnosis, tumour size at diagnosis, and survival probability for the early stage of liver cancer.

## Materials and methods

Study patients diagnosed with liver cancer were selected from the SEER 18 Regs custom data with additional treatment fields from 1 January 2004 to 31 December 2015 based on the Derived American Joint Committee on Cancer (AJCC) stage Group, 6th ed. Insurance status was grouped as ‘Medicaid’, ‘Insured’, ‘Uninsured’, ‘Unknown’. Location was grouped as ‘Metro (Metropolitan)’, ‘Rural (living in farming or country life)’, ‘Urban (located in a city or city life)’, and region was grouped as ‘North’, ‘Southwest’, ‘South’. Informed consent for participation in the study has been obtained from SEER datasets. The SEER database was publicly available and the private data of all patients have been eliminated from the SEER database. The study was approved by the Ethics Committee of Shanghai Tenth People’s Hospital, Tongji University School of Medicine (SHSY-IEC-P-15-19).

The inclusion criteria were as follows: (1) diagnosed as HCC; (2) primary tumour location was in the liver; (3) known cause of death; (4) complete treatment information. And the exclusion criteria were as follows: (1) metastatic liver cancer or other cancers; (2) incomplete information of treatment; (3) death caused by other cancers; and (4) unknown cause of death.

Patients diagnosed with stage I and all other stages of liver cancer were compared based on cohort clinical characteristics. Both *t*-tests and Fisher exact tests were performed to acquire *p* values for continuous and categorical variables, correspondingly. All tests were two-sided. Furthermore, odds ratios (OR) were analysed. The age-adjusted incidence rates for different stages of liver cancer were obtained using SEER*stat (v8.3.9) and standardized using the 2000 US population. Incidence trends and their corresponding annual percent changes (APC) of liver cancer were estimated by Joinpoint software (v4.9.0.0). Moreover, the age-adjusted incidence rates were calculated for subgroups within stage I liver cancer of chemotherapy, race, Hispanic ethnicity, sex, region, location, and radiation. The relationship between median tumour size over years of diagnosis was evaluated using linear regression with interaction analysis for all stages of liver cancer. This article considered a *p* value of <.05 as statistically significant.

Independent group *T*-tests were performed to acquire the mean ages, *p*-value, and confidence interval for different stages of liver cancer. Survival trends over year were simulated using the Kaplan–Meier curve models.

## Results

### Baseline characteristics

Table 1 demonstrates the baseline characteristics of stage I vs. other stages of liver cancer diagnosed over the study period. Those diagnosed with stage I liver cancer were more likely to be females (27.96% vs. 24.5%; *p* < .001). Patients who were married are 2.41 times more likely to be in stage I than otherwise (OR = 2.41, CI = 2.33–2.48, *p* < .0001), while stage I patients are more probably to be married (55.88% vs. 44.12%; *p* < .001). Similarly, patients who lived in metro areas were 1.11 times more likely to be in stage I than patients who lived in urban and rural areas (OR = 1.11, CI = 1.04–1.67, *p* < .0005).

**Table 1.** Characteristics of cases in SEER registries diagnosed with liver cancer from 2004 to 2015.

Characteristic	Stage I (*n* = 23 615)	All other staged cancers (*n* = 55 765)
Mean age at diagnosis, year (SD)	64.4 (11.6)	64.5 (12.2)
Sex, *n* (%)		
Female	6603 (27.96)	13 665 (24.50)
Male	17 012(72.04)	42 100 (75.50)
Race, *n* (%)		
American Indian/Alaska Native	289 (1.22)	735 (1.32)
Asian or Pacific Islander	3966 (16.79)	8438 (15.13)
Black	2897 (12.27)	7568 (13.57)
Unknown	100 (0.42)	191 (0.34)
White	16 363 (69.29)	38 833 (69.64)
Hispanic ethnicity, yes (%)	4557 (19.30)	10 474 (18.78)
Location, *n* (%)		
Metro	21 595 (91.60)	50 510 (90.70)
Rural	175 (0.74)	514 (0.92)
Urban	1806 (7.66)	4667 (8.38)
Region, *n* (%)		
North	6521 (27.61)	15 719 (28.00)
Southwest	12 279 (52.0)	28 299 (50.75)
South	4815 (20.39)	11 747 (21.07)
Martial status, married/un-married (%)	12 534/9895 (55.88)	27 894/52 973 (52.66)
Insurance status, *n* (%)		
Uninsured	530 (2.68)	1988 (4.47)
Medicaid	4510 (22.83)	9684 (21.79)
Insured	14 321 (72.52)	27 474 (61.81)
Unknown	390 (1.97)	5301 (11.93)
Year of diagnosis, *n* (% of each year)		
2004	1199 (5.08)	3551 (6.37)
2005	1263 (5.35)	3771 (6.76)
2006	1402 (5.94)	3996 (7.17)
2007	1540 (6.52)	4266 (7.65)
2008	1637 (6.93)	4488 (8.05)
2009	1859 (7.87)	4760 (8.54)
2010	2046 (8.66)	4670 (8.37)
2011	2200 (9.32)	4922 (8.83)
2012	2466 (10.44)	5031 (9.02)
2013	2521 (10.68)	5325 (9.55)
2014	2677 (11.34)	5521 (9.90)
2015	2805 (11.88)	5464 (9.80)
Median tumour size, cm (range)	3.5 (0.1–10)	5.6 (0–9.96)
Neoadjuvant radiation, received/not received (%)	107/23 508 (0.45/99.55)	301/55 461 (0.54/99.45)
Chemotherapy, received/not received (%)	8671/14 944 (36.72/63.28)	18 419/37 346 (33.03/66.97)

*p* values compared between cases with stage I disease and all other staged cases were < .001 for all variables except for radiation (*p* = .1284) and Hispanic ethnicity (*p* = .0921).

In clinic, patients with early-stage liver cancer are usually treated by surgery. Common surgical methods, such as liver transplantation and hepatectomy, can quickly and effectively help patients improve the status of the focus and improve the survival rate. However, for liver cancer patients with metastasis to other organs in the late stage, the surgical effect is often poor after the focus involves multiple parts, so chemotherapy can be considered clinically to help patients improve their symptoms. In SEER database, some liver cancer patients received chemotherapy. To evaluate whether patients with early-stage liver cancer can benefit from chemotherapy, the age-adjusted incidence rates were calculated for subgroups within stage I liver cancer of chemotherapy.

Stage I liver cancers were more likely to receive chemotherapy (32.01% vs. 28.58%; *p* < .001), while those received chemotherapy are 1.18 times more likely to be in stage I than otherwise (OR = 1.18, CI = 1.14–1.21, *p* < .0001). Moreover, stage I cases were more probably to have medical insurance (76.05% vs. 73.94%; *p* < .0001), while those had medical insurance were 1.12 times more likely to be in stage I than otherwise (OR = 1.12, CI = 1.08–1.67, *p* < .0001).

### Trends in incidence

**Figure 1.**
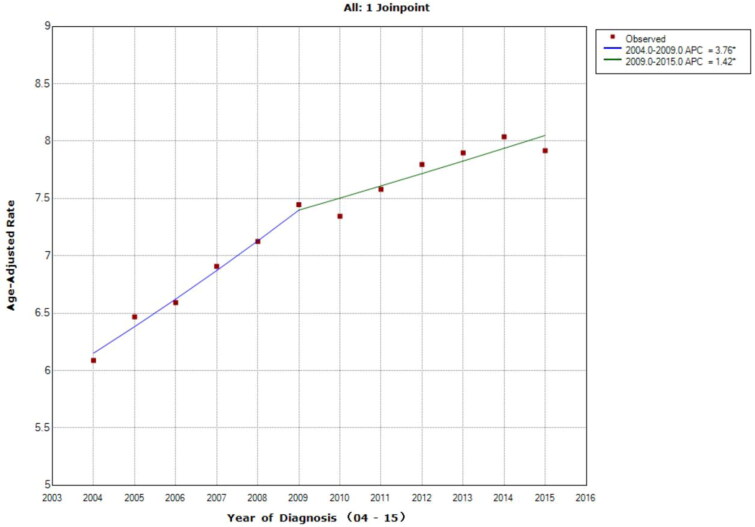
Age-adjusted incidence trends over time for liver cancer, shown as age-adjusted incidence rate per 100k over time (2004–2015). The overall incidence of liver cancer increased from 2004 to 2015, with a greater increase from 2004 to 2009. The APC for all stages of liver cancer was 2.5. Asterisk denotes annual percent change (APC) with two-sided *p* < .05.

**Figure 2.**
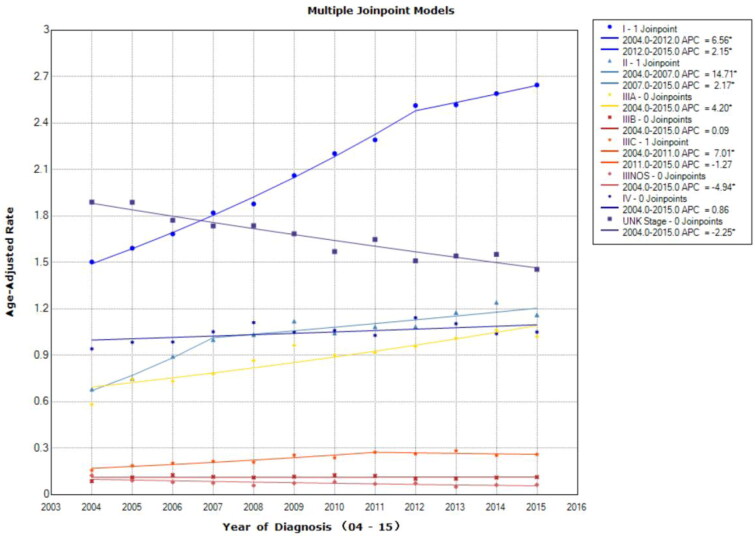
Age-adjusted incidence trends over time for stage I–IV liver cancer, shown as age-adjusted incidence rate per 100k. From 2004–2012, it increased the most, following a smaller increase from 2012 to 2015. The APC of stage I cases was more than twice of the APC for all stages. Asterisk denotes annual percent change (APC) with two-sided *p* < .05.

The overall incidence of liver cancer increased from 2004 to 2015, with a greater increase from 2004 to 2009 (APC = 3.8, 95% CI = 2.6–5; *p* < .001; 2009–2015 APC = 1.4, 95% CI = 0.7–2.2; *p* = .003; Figure 1). The APC for all stages of liver cancer was 2.5 (95% CI = 1.9–3; *p* < .1; Figure 1). Stage I cases increased most tremendously. From 2004–2012, it increased the most (APC = 6.6, 95% CI = 6.1–7; *p* < .001), following a smaller increase from 2012 to 2015 (APC = 2.2, 95% CI = 0.5–3.8; *p* = .018). The APC of stage I cases was more than twice of the APC for all stages (APC = 5.3, 95% CI = 4.9–5.8; *p* < .1; Figure 2).

From 2004 to 2007, the incidence of stage II had the greatest temporal growth (APC = 14.7, 95% CI = 4.9–25.4; *p* = .008), following a lesser increase from 2007 to 2015 (APC = 2.2, 95% CI = 0.6–3.7; *p* = .012). Incidence of stage IIIA cases increased moderately (APC = 4.2, 95% CI = 2.9–5.5; *p* < .001). From 2004 to 2011, incidence of stage IIIC cases indicated similar trends (APC = 7.0, 95% CI = 3.7–10.4; *p* = .001), but then decreased from 2011 to 2015 (APC = −1.3, 95% CI = −7 to 4.8; *p* = .63). Conversely, stage IIINOS diagnosis decreased the most (APC = −4.9, 95% CI = −7.6 to −2.2; *p* = .003), while incidence of UNK stage decreased as well (APC = −2.3, 95% CI = −2.7 to −1.8; *p* < .001). Stage IV diagnoses had the smallest increase (APC = 0.9, 95% CI = 0–1.7; *p* = .053; Figure 2).

Incidence trend among different subgroups of stage I liver cancer was analysed. Stage I cases across all races showed an increase in incidence trend. The increase was most evident in American Indians/Alaska Natives (APC = 8.1, 95% CI = 4.3–12; *p* = .001). Similar trends can be observed for black race cases (APC = 4.9, 95% CI = 3.9–5.9; *p* < .001). From 2004 to 2012, white race cases demonstrated a considerable increase (APC = 7.7, 95% CI = 6.9–8.5; *p* < .001), following a smaller increase from 2012 to 2015 (APC = 1.8, 95% CI = −0.9 to 4.5, *p* = .16; Supplementary Figure S1).

**Figure 3.**
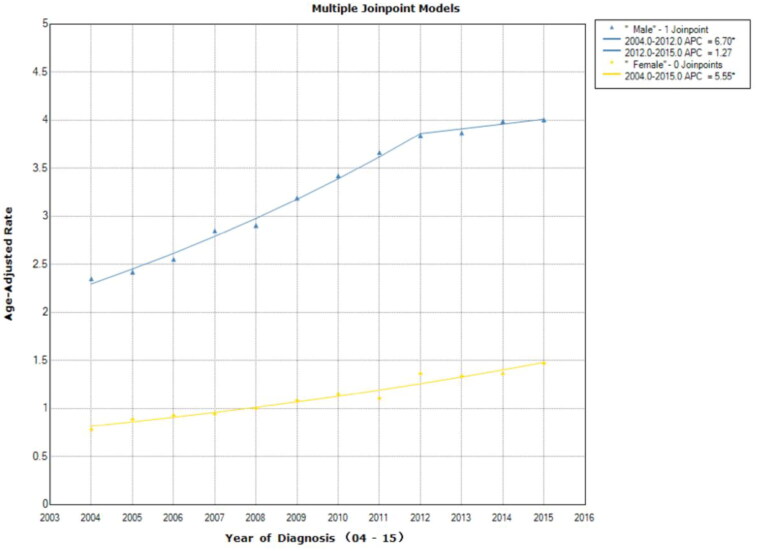
Age-adjusted incidence trends over time for Stage I liver cancer according to sex. From 2004 to 2012, male diagnoses had the greatest rise, but then levelled off from 2012 to 2015. Female cases showed an increase throughout the study period. Asterisk denotes annual percent change (APC) with two-sided *p* < .05.

**Figure 4.**
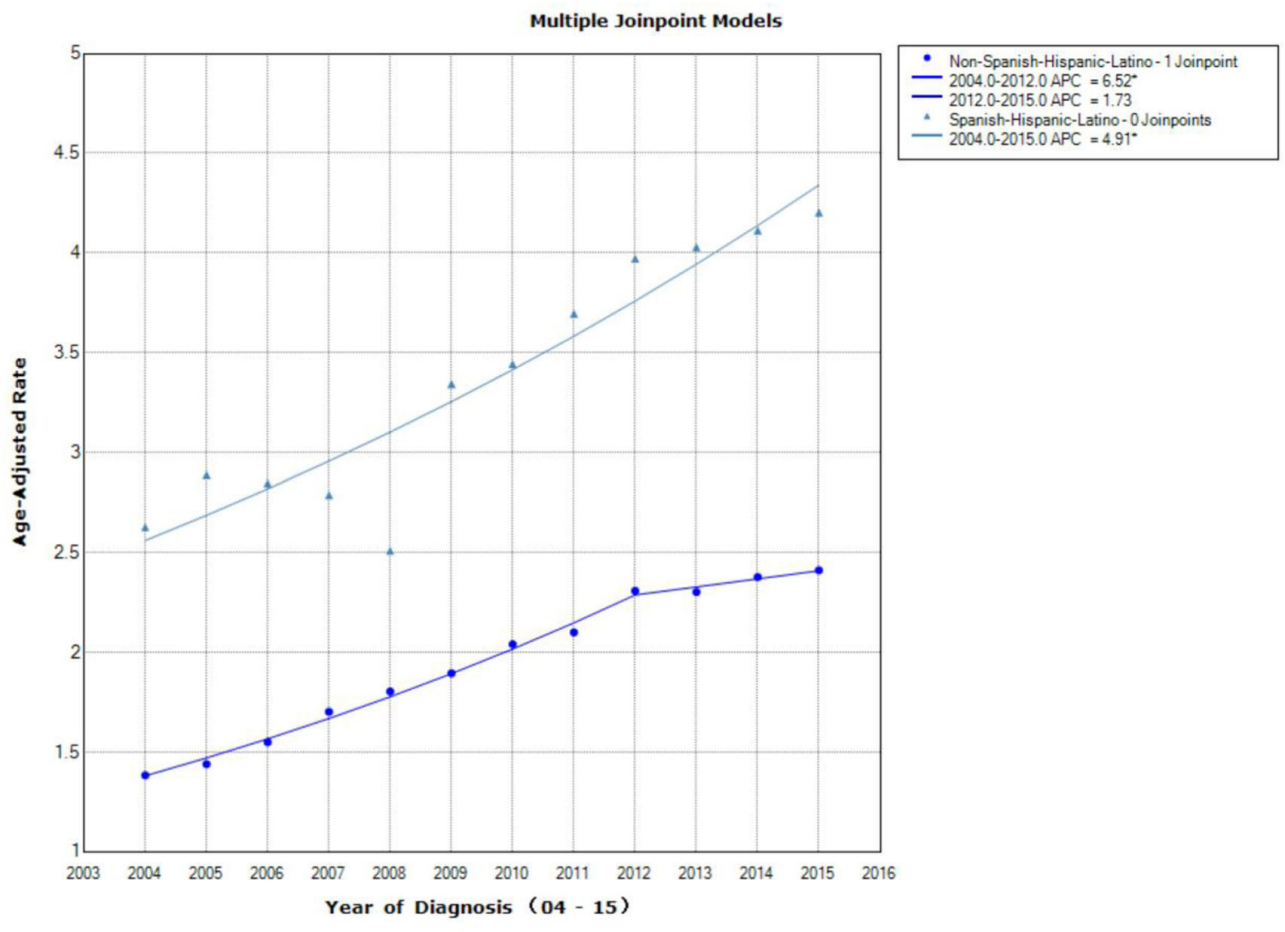
Age-adjusted incidence trends over time for Stage I liver cancer according to Hispanic ethnicity. A greater increase can be observed for non-Spanish–Hispanic–Latino diagnoses from 2004 to 2012; from 2012 to 2015 the trend levelled off. Asterisk denotes annual percent change (APC) with two-sided *p* < .05.

From 2004 to 2012, male diagnoses had the greatest rise (APC = 6.7, 95% CI =6–7.4, *p* < .001), but then levelled off from 2012 to 2015 (APC = 1.3, APC = −1.2 to 3.8; *p* = .265). Female cases showed an increase throughout the study period (APC = 5.6, 95% CI = 4.7–6.4, *p* < .001; Figure 3). Spanish–Hispanic–Latino showed an increase in incidence trend (APC = 4.9, 95% CI = 3.6–6.3; *p* < .001). A greater increase can be observed for non-Spanish–Hispanic–Latino diagnoses from 2004 to 2012 (APC = 6.5, 95% CI = 5.9–7.2; *p* < .001); from 2012 to 2015 the trend levelled off (APC = 1.7, 95% CI − 0.5 to 4; *p* = .113; Figure 4). Both female and male cases demonstrated an increase in incidence, which grow from 2004 to 2012 (APC = 6.6, 95% CI = 6.1–7; *p* < .001), following a smaller increase from 2012 to 2015 (APC = 2.2, 95% CI = 0.5–3.8; *p* = .018).

Incidence rate of stage I liver cancer raised across all regions. Northern case had the greatest increase (APC = 6.9, 95% CI = 4.7–9.3; *p* < .001). Southern cases demonstrated a similar increase (APC = 5.1, 95% CI = 2.6–7.6; *p* = .001). From 2004 to 2011, incidence for Southwest cases increased considerably (APC = 6.6, 95% CI = 4.6–8.5; *p* < .001), following a smaller increase from 2011 to 2015 (APC = 1.7, 95% CI = −1.7–5.1; *p* = .284). Furthermore, incidence of cases diagnosed in urban and rural areas increased significantly (APC = 7.4, 95% CI = 4.8–10; *p* < .001 and APC = 7.3, 95% CI = 0.8–14.1, *p* = .03, respectively). From 2004 to 2012, incidence in metro areas raised (APC = 6.8, 95% CI = 5.9–7.7; *p* < .001), following a smaller increase from 2012 to 2015 (APC = 2.5, 95% CI = −0.5–5.6, *p* = .094; Supplementary Figure S2).

**Figure 5.**
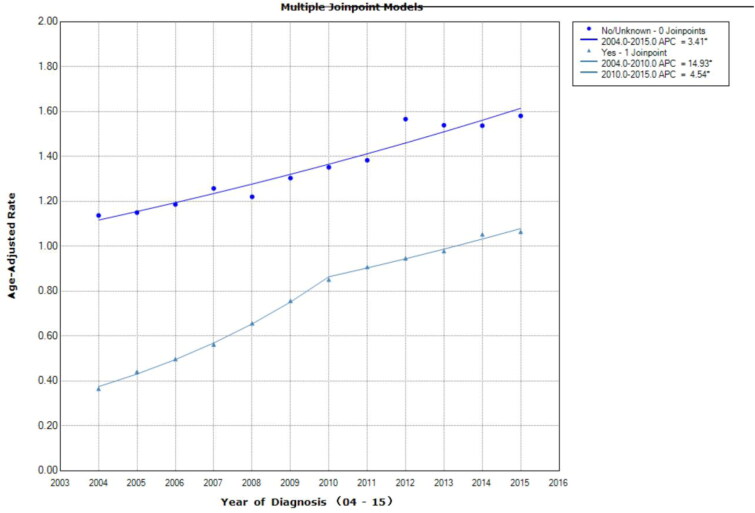
Age-adjusted incidence trends over time for Stage I liver cancer according to whether receiving chemotherapy. Rise in incidence rates was observed for all stage I cases no matter if they receive chemotherapy or neoadjuvant radiation. Besides, the increase in incidence for cases receiving chemotherapy was almost five times higher than cases not receiving chemotherapy from 2004 to 2010, but then levelled off from 2010 to 2015. Asterisk denotes annual percent change (APC) with two-sided *p* < .05.

Rise in incidence rates was observed for all stage I cases no matter if they receive chemotherapy or neoadjuvant radiation (Supplementary Figure S3; Figure 5). Incidence of cases not receiving neoadjuvant radiation increased during the study period (2004–2012 APC = 6.6, 95% CI = 6.1–7.1; *p* < .001, 2012–2015 APC = 2.0, 95% CI = 0.3–3.7; *p* = .025). Incidence for cases received neoadjuvant radiation was three-fold higher than cases not receiving it (APC = 15, 95% CI = 7.3–23.2; *p* = .001, Supplementary Figure S3). Similarly, the increase in incidence for cases receiving chemotherapy was almost five times higher than cases not receiving chemotherapy from 2004 to 2010 (Chemotherapy yes: APC = 14.9, 95% CI = 13.7–16.2; *p* < .001; Chemotherapy no: APC = 3.4, 95% CI = 2.8–4; *p* < .001), but then levelled off from 2010 to 2015 (APC = 4.5, 95% CI = 3.5–5.6; *p* < .001, Figure 5).

### Median tumour size at diagnosis

**Figure 6.**
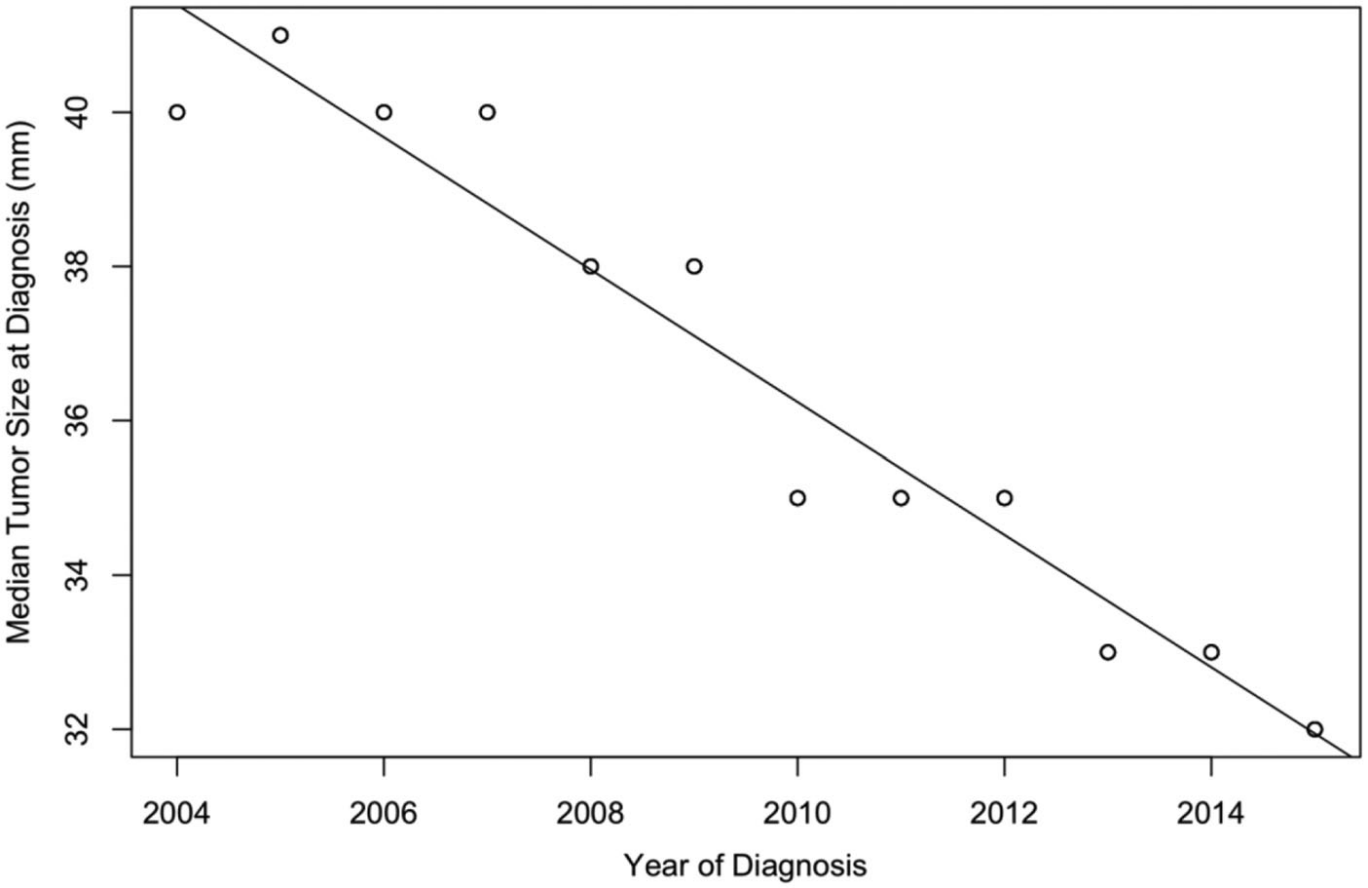
Trends of median tumour size over year of diagnosis for stage I liver cancer. As the year of diagnosis became recent, the tumour size of stage I cases decreased.

All stages of median tumour size at diagnosis demonstrated a statistically significant linear relationship with time. As the year of diagnosis became recent, the tumour size of stage I cases decreased (*P* = 2.2E–16, Figure 6). The reduction in tumour size is also shown for stage II, IIIA, IIINOS, and UNK stages. Conversely, median tumour size for stage IIIB, IIIC, and IV increased.

### Trends in mean age at diagnosis

**Figure 7.**
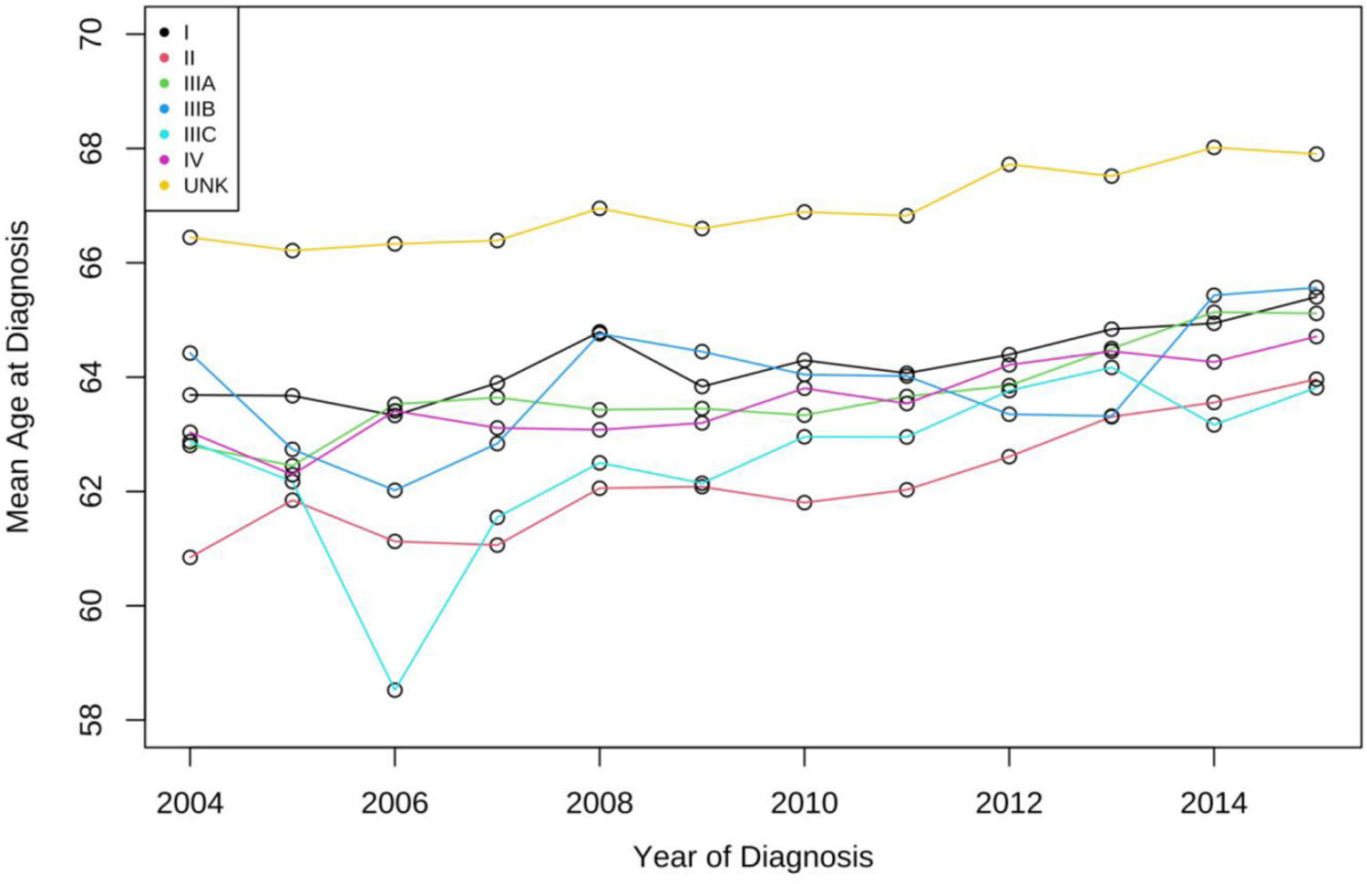
Trends in mean age at diagnosis for stage I–IV liver cancer. The mean age of Stage I liver cancer increased by 1.72 years from 2004 to 2015, while the mean age of stage IV increased by 1.67. Similar trends can be observed in stage IIIA and UNK stage as well, with an increase of 2.31 and 1.46, respectively.

The overall trend of mean age at diagnosis increased by 1.57 ages (95% CI = 1.10–2.04; *P* = 4.12E-11). Moreover, all stages showed an increasing trend. The mean age of stage II Liver cancer demonstrated the most increase of 3.12 (95% CI = 2.12–4.11; *P* = 1.07E-9); The mean age of stage I liver cancer increased by 1.72 years from 2004 to 2015 (95% CI = 0.93–2.50; *P* = 2.01E-5), while the mean age of stage IV increased by 1.67 (95% CI = 0.50–2.85; *p* = .005). Similar trends can be observed in stage IIIA and UNK stage as well, with an increase of 2.31 (*p* < .001, 95% CI = 1.11–3.51) and 1.46 (*p* < .001, 95% CI = 0.52–2.39; Figure 7), respectively.

### Trends in survival

The overall 5-year survival probability of liver cancer decreased, from 96.4% (95% CI = 95.3–97.6%) in 2004 to 82.9% (95% CI = 80.9–84.9%). The survival rates for stages I, II, IIIA, IIIC, and UNK were generally stable with slight decrease from 2004 to 2010, and then decreased sharply from 2010 to 2011.

For cases diagnosed in 2004, stage I was less risky than stage II (HR = 0.94, CI = 0.73–1.2; *p* < .001); while it became almost as risky as stage II for cases diagnosed in 2011 (HR = 1.05, 95% CI = 0.92–1.20; *p* < .001). During the study period, stage I became riskier over time than stage IIIB (HR = 1.02, 95% CI = 0.44–2.38; *p* < .001 to HR = 1.19, 95% CI = 0.70–2.01; *p* < .001), and similar trends can be observed for stage IIIC as well. Stage I was 0.26 times less risky than stage IV in 2004 (HR = 0.26, 95% CI = 0.12–0.56; *p* < .001); however, it became 0.58 times less risky than stage IV in 2011 (HR = 0.58, 95% CI = 0.35–0.96; *p* < .001).

**Figure 8.**
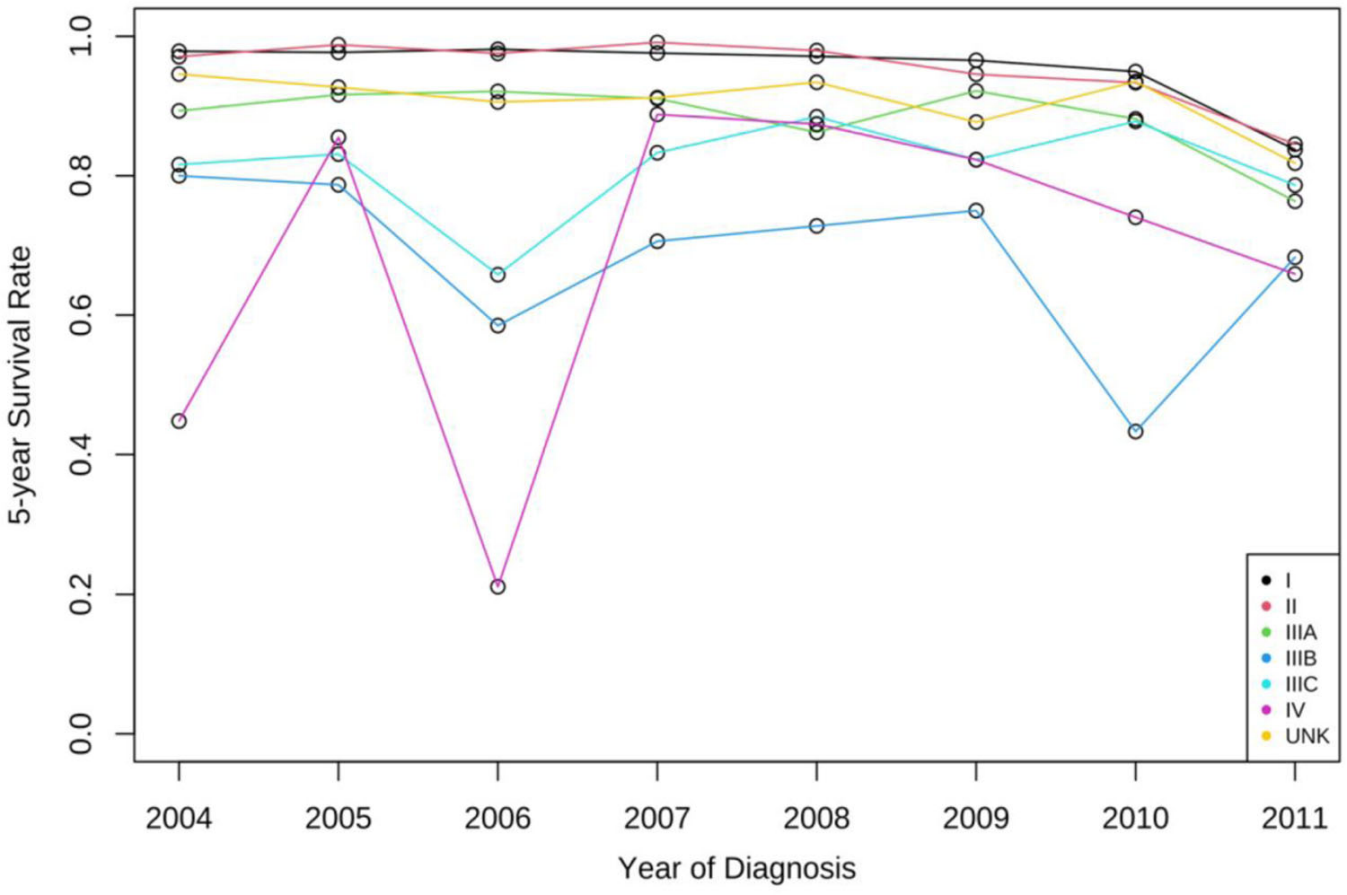
Trends in 5-year survival probabilities for stage I–IV liver cancer. The 5-year survival probability for stage IV increased from 2004 to 2005, following a sharp decrease in 2006 and then bounces back in 2007, while both stages IIIB and IIIC demonstrated a similar trend. Survival rate for stages such as stage II, stage IIIA, stage IIIB, and stage IIIC all decreased.

Moreover, from 2004 to 2011, the 5-year-overall survival for stage I liver cases worsened from 97.9% (95% CI = 96.4–99.3%) to 83.7% (95% CI = 81.2–0.86.3%). Stage IV is the only stage that had a greater survival probability in 2011 (65.9%, 95% CI = 47.7–91.2%) than 2004 (44.8% 95% CI = 21.9–91.9%). The 5-year survival probability for stage IV increased from 2004 to 2005, following a sharp decrease in 2006 and then bounces back in 2007, while both stages IIIB and IIIC demonstrated a similar trend. Survival rate for stages such as stage II, stage IIIA, stage IIIB, and stage IIIC all decreased. For stage I cases, reductions in 5-year survival probabilities were shown among groups defined by sex and race. However, survival probabilities dropped rapidly if the individual received chemotherapy. Survival probability also reduced if the individual was diagnosed in the North compared with South and Southwest (Figure 8). Furthermore, 10-year survival probability for stage I cases worsened from 97.3% in 2004 (95% CI = 95.5–99.1%) to 79.6% in 2006 (95% CI = 75.1–84.5%).

## Discussion

According to our findings, the age-adjusted incidence rate of liver cancer increased from 2004 to 2015, and the incidence of stage I cases was significantly higher than other stages (Figures 1 and 2). Since this significant amount of change was particular for stage I and occurred in a short period of time, it is unlikely to be the result of biological factors. This is likely the result of early diagnosis, improved surveillance for individuals with existing liver diseases, and better insurance coverage.

We found that the mean age at diagnosis for all stages of liver cancer increased by 1.57 years, which seems to be a global trend [10]. The overall mean age of stage II liver cancer increased by 3.12 years, while stage I liver cancer increased by 1.72 years (Figure 7). This could be explained by lower incidence rate among younger population due to the promotion of the Hepatitis B vaccine; better medical treatment for patients with existing liver disease, which delays or prevents the onset of liver cancer among the youngers; and increased incidence of fatty liver disease among the elders [11]. Prevalence and improvements of diagnostic imaging tests such as ultrasound, CT, and MRI might contribute to the early diagnosis of stage I liver cancer as well.

Subgroups among stage I liver cancer cases were analysed. The age-adjusted incidence rate for men is much higher than women (Figure 3). This is consistent with past findings since most cancer types have more male cases [12].

Moreover, socioeconomic status, income, and accessibility of health care services are all potential factors that result in the difference of incidence rate in subgroups of stage I liver cancer. The age-adjusted incidence rate differs across race groups. Asian cases have a higher incidence rate, but the slowest APC across time. American Indian/Alaska native cases have a relatively lower incidence rate with the most rapid APC over time. White race cases have the lowest incidence rate, but its increase in incidence rate is considerable (Supplementary Figure S1). The ethnic differences in liver cancer incidence in the United States suggest the racial disparities and differences in accessibility of health care. This difference in incidence rate across races is possibly caused by disparities in socioeconomic status [13, 14].

Our findings across locations and regions of stage I cases further strengthen our suggestions. Cases living in the south of the United States have the lowest incidence rate and slowest change in incidence of stage I liver cancer, while cases living in the southwest have the highest. The greatest increase in incidence rate appears in the north, while the southwest shares a similar trend. Cases living in metro areas have the highest incidence rate but the slowest change, while cases living in rural areas have the lowest incidence rate but faster increase (Supplementary Figure S2). This is likely the result of the difference in mean income and disparities in health care services provision across different locations since higher income and prevalence of health care services in metro areas facilitate the early diagnosis of liver cancer [15].

The liver cancer patients not receiving neoadjuvant radiation had a significantly higher incidence rate; however, the incidence rate for those receiving neoadjuvant radiation had a faster APC (Supplementary Figure S3). The incidence rate was lower for those who did receive chemotherapy. Those who receive chemotherapy showed a greater APC. Conversely, those who did not receive chemotherapy had a higher incidence rate, but much slower change (Figure 5).

The overall 5-year survival probability for liver cancer decreased. However, the survival rate for the early stages of liver cancer remained high. Particularly, the 5-year survival rate for stages I, II, IIIA, and UNK were generally stable with a slight decrease, and stage IIIB, IIIC, and IV fluctuated the most (Figure 8). This indicates the need for regular screening and consultation of risk factors for high-risk patients. The relatively poor prognosis shown in the late stages of liver cancer during the past decade demonstrates the greater need to diagnose liver cancer at an early stage to improve survival probability.

Although the survival rate of liver cancer has improved in recent years, the prognosis of liver cancer patients is still not optimistic. In the United States, >60% of liver cancer patients are caused by modifiable risk factors. For example, according to statistics of medical insurance personnel, 32% of liver cancer patients are caused by metabolic disorders. A small number are due to hepatitis C virus infection, alcohol abuse, and smoking. And the proportion of patients caused by hepatitis B virus infection is <5%. On the other hand, according to the development speed of the latest precision medicine and immunotherapy, the survival rate of cancer will be further improved. But the biggest challenge now is still how to make more patients benefit from these new therapies. Therefore, the risk factors composed of gender, age, country, and race/religion and the differences in the beneficiaries of emerging treatments account for most of the variables of liver cancer mortality.

Our study has several limitations. TNM staging of liver cancer is one of the important clinical features of liver cancer patients. TNM staging of liver cancer has an important impact on the choice of treatment scheme and operation mode of liver cancer patients, as well as the prognosis of liver cancer patients. Liver function and performance status also affect treatment and prognosis of liver cancer patients. Besides, there are some differences in staging of liver cancer between countries. In this study, patients diagnosed with liver cancer were selected from the SEER 18 Regs custom data based on the AJCC stage group, 6th ed. When we extend this research result to other countries or regions for horizontal comparison, definition of the staging of liver cancer is necessary. Moreover, in this study, only data from 2004 to 2015 had been used, which is not very recent and has geographical limitations. SEER also lacks covariate data that could facilitate more complex analysis.

## Conclusion

The incidence trend for stage I liver cancer had increased during the study period with an increase in mean age at diagnosis. The survival probability of early stages of liver cancer remained high, with a slight decrease over the study period. This further strengthened the need to better surveillance of high-risk populations and diagnosis of liver cancer at an early stage.

## Supplementary Material

Supplemental Material

## Data Availability

The datasets supporting the conclusions of this article are included within the article.

## Abbreviations

AJCC: American Joint Committee on Cancer
APC: annual percent changes
CI: confidence interval
OR: odds ratio
UNK: unknown stage
SEER: Surveillance, Epidemiology and End Results.
