# Supplementary material for: Recent trends in the incidence and survival of stage I liver cancer: a surveillance, epidemiology, and end results analysis
Source: Ann Med. 2022 Nov 12;54(1):2785–95. doi: 10.1080/07853890.2022.2131328 (PMC9662040; doi:10.1080/07853890.2022.2131328)
Supplement: Supplemental Material [file IANN_A_2131328_SM9914.docx]

**SUPPLEMENTAL INFORMATION**

**Recent Trends in the Incidence and Survival of Stage I Liver Cancer: A Surveillance, Epidemiology, and End Results Analysis**

Supplemental information contains 3 supplemental Figures and legends.


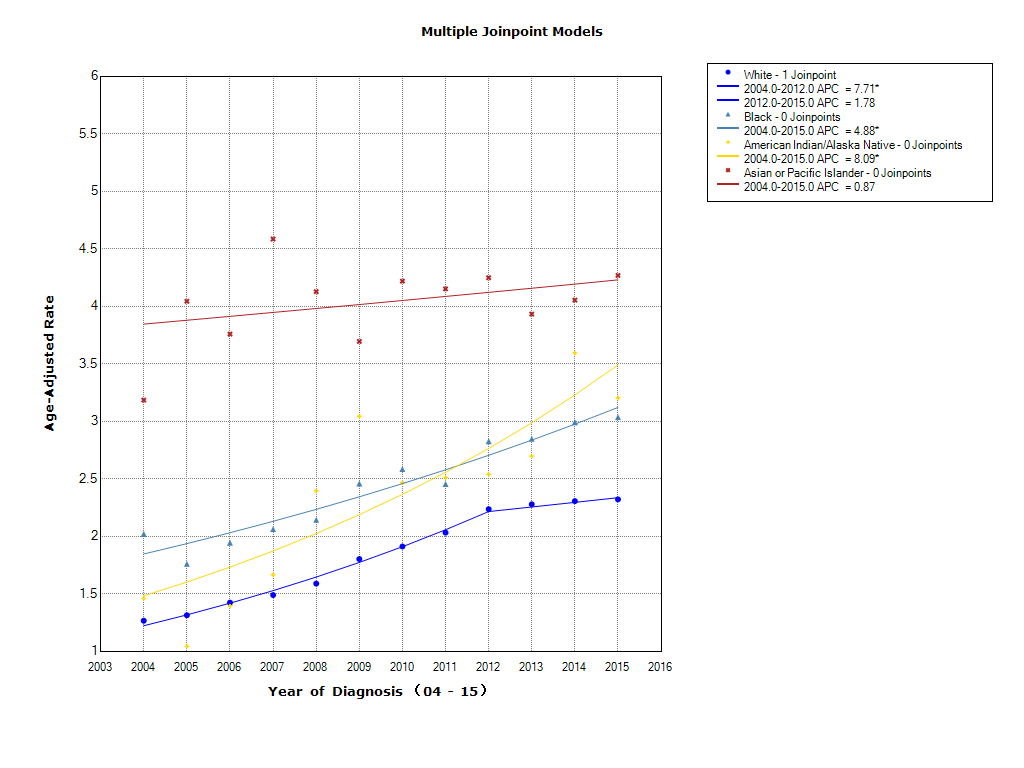
Figure S1. Age-adjusted incidence trends over time for Stage I liver cancer according to race. Asterisks denotes annual percent change (APC) with two-sided P < 0.05.


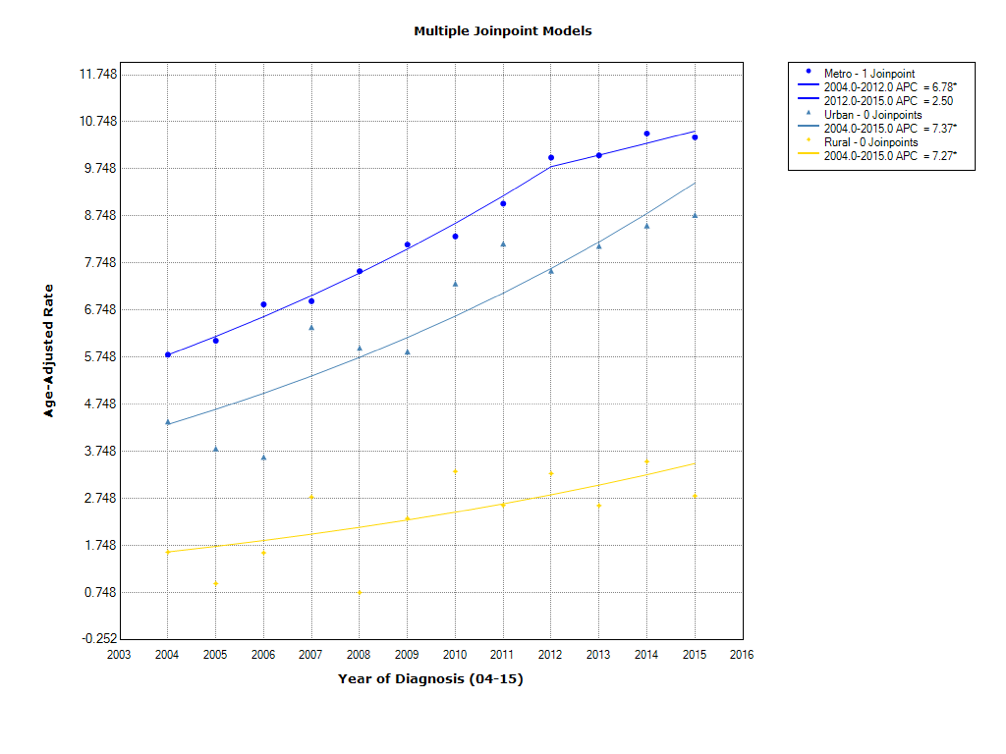


Figure S2. Age-adjusted incidence trends over time for Stage I liver cancer according to location. Asterisks denotes annual percent change (APC) with two-sided P < 0.05.


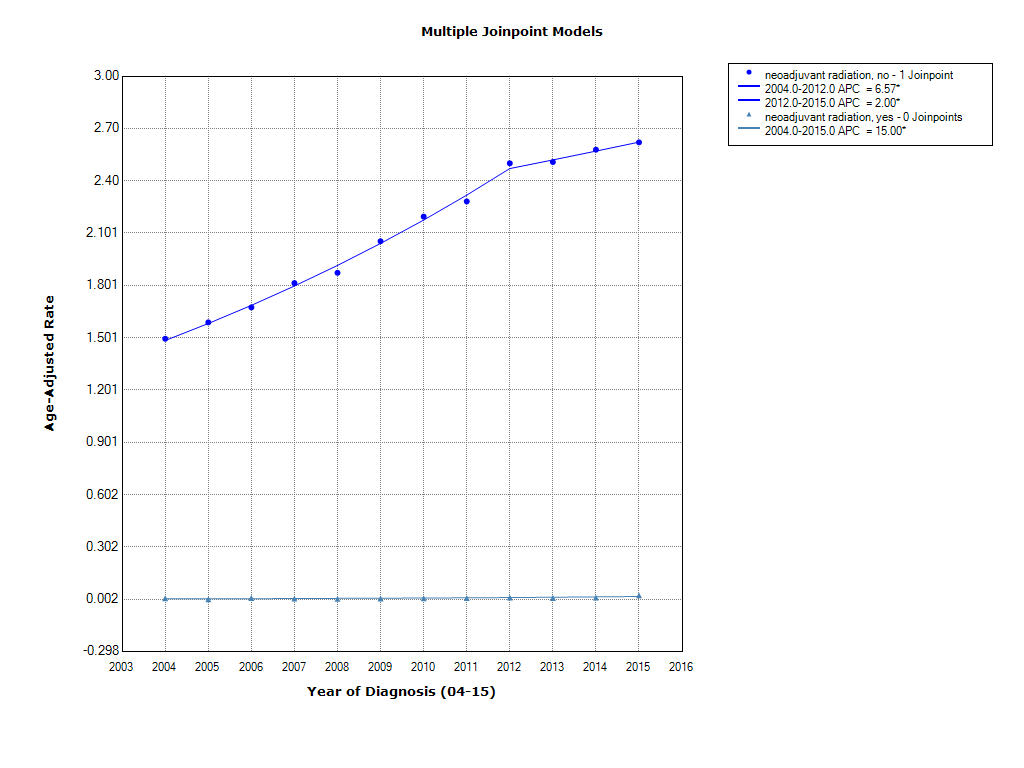


Figure S3. Age-adjusted incidence trends over time for Stage I liver cancer according to whether receiving neoadjuvant radiation. Asterisks denotes annual percent change (APC) with two-sided P < 0.05.
